# Detecting Sex-Related Changes to the Metabolome of a Critically Endangered Freshwater Crayfish During the Mating Season

**DOI:** 10.3389/fmolb.2021.650839

**Published:** 2021-04-16

**Authors:** Emily D. Lette, Quinton F. Burnham, Nathan Lawler, Pierre Horwitz, Mary C. Boyce, David I. Broadhurst, Rodney Duffy, Annette Koenders

**Affiliations:** ^1^Centre for Ecosystem Management, Edith Cowan University, Perth, WA, Australia; ^2^Centre for Integrative Metabolomics and Computational Biology, Edith Cowan University, Perth, WA, Australia; ^3^Department of Primary Industries and Regional Development of Western Australia, Perth, WA, Australia

**Keywords:** captive breeding, *Cherax cainii*, *Cherax tenuimanus*, conservation, haemolymph, LC-MS, oxidative stress, reproduction

## Abstract

Captive breeding is a vital tool in the conservation of highly endangered species, as it is for the Margaret River hairy marron, *Cherax tenuimanus*, from the south west of Australia. A close relative, *Cherax cainii*, has almost completely displaced *C. tenuimanus* in the wild and is a successful aquaculture species, whereas *C. tenuimanus* has performed poorly in captivity. We used untargeted liquid chromatography-mass spectrometry to obtain metabolomic profiles of female and male *C. tenuimanus* held in controlled aquarium conditions during their reproductive period. Using repeated haemolymph sampling we tracked the metabolomic profiles of animals just prior to and for a period of up to 34 days after pairing with a similar sized potential mate. We identified 54 reproducible annotated metabolites including amino acids, fatty acids, biogenic amines, purine and pyrimidine metabolites and excretion metabolites. Hierarchical clustering analysis distinguished five metabolite clusters. Principal component-canonical variate analysis clearly distinguished females from males, both unpaired and paired; similar trends in profile changes in both sexes after pairing; and a striking shift in males upon pairing. We discuss three main patterns of metabolomic responses: differentiation between sexes; reactive responses to the disturbance of pairing; and convergent response to the disturbance of pairing for males. Females generally had higher concentrations of metabolites involved in metabolic rate, mobilisation of energy stores and stress. Responses to the disturbance of pairing were also related to elevated stress. Females were mobilising lipid stores to deposit yolk, whereas males had a rapid and strong response to pairing, with shifts in metabolites associated with gonad development and communication, indicating males could complete reproductive readiness only once paired with a female. The metabolomic profiles support a previously proposed potential mechanism for displacement of *C. tenuimanus* by *C. cainii* in the wild and identify several biomarkers for testing hypotheses regarding reproductive success using targeted metabolomics.

## Introduction

Freshwater crayfish are highly speciose, with over 640 species worldwide including 148 species in Australasia (Crandall and Buhay, 2008; Coughran and Furse, 2012). They are ecologically significant as bioindicators, keystone species, ecosystem engineers, and umbrella species (Brown and Lawson, 2010; Horwitz, 2010; Coughran and Furse, 2012; Richman et al., 2015) as well as a valuable human food source (Piper, 2000; FAO, 2018). Despite their significance, one-third of freshwater crayfish species are classified as threatened under IUCN criteria from a range of processes including climate change, habitat loss or modification, overfishing, pollution and environmental toxins, biological invasion, displacement by introduced species, hybridisation, and the spread of pathogens (Horwitz, 2010; Richman et al., 2015; Westhoff and Rosenberger, 2016).

The south-west of Australia hosts six species of endemic freshwater crayfish in the widespread genus *Cherax* with two large species commonly referred to as ‘marron’. *Cherax tenuimanus* Smith (1912) is geographically restricted to the Margaret River, and is referred to as ‘Margaret River hairy marron’ to differentiate them from ‘smooth marron’, *Cherax cainii* Austin, 2002 (Austin and Ryan, 2002) with a wide range (Bunn et al., 2008). *C. cainii* have been translocated multiple times throughout the south-west of Australia and other areas in Australia for the purpose of aquaculture with production at around 60 tonnes per annum (Stanley, 2016) and recreational fishing (Morrissy, 1992; Beatty et al., 2016), because they breed successfully in captivity. One of these locations is the Margaret River, where *C. cainii* are displacing *C. tenuimanus*, possibly because the former has higher fecundity and starts reproduction earlier than the latter (Austin and Ryan, 2002; Bunn et al., 2008; Duffy et al., 2014). *C. tenuimanus* is now only found in the upper three pools in the Margaret River (Austin and Bunn, 2010; Duffy et al., 2014) with fewer than 400 individuals remaining in the wild (R. Duffy, unpublished). Consequently *C. tenuimanus* is listed as Critically Endangered under the Western Australian Wildlife Conservation Act 1950 [Wildlife Conservation (Specially Protected Fauna) Notice 2015], nationally under the Environment Protection and Biodiversity Conservation Act 1999, List of Threatened Fauna of Australia, as well as Critically Endangered under the IUCN red list of threatened species criteria since 2010 (Austin and Bunn, 2010).

An *ex situ* captive breeding program for *C. tenuimanus* has been established using best practice techniques developed for *C. cainii* (Duffy et al., 2014; Duffy and Day, 2015) but despite this, breeding output has been low and declining (R. Duffy, unpublished). The reasons for the breeding failure are unknown, and we hypothesis that documentation of the metabolomic profiles will lead to identification of biomarkers of various physiological processes and ultimately can attribute underlying causes for differences in fecundity. If temperature and day length (Morrissy, 1970; Huner, 1994) and the presence of the opposite sex are environmental cues required to prepare for mating, then metabolites should be able to pinpoint aspects of the reproductive process where *ex situ* conservation efforts can focus.

Whilst still new to the aquaculture industry and captive breeding of aquatic organisms (Alfaro and Young, 2016), metabolomics has been used to assess the health of stock by identifying biomarkers for stressors to environmental conditions for aquatic organisms, including decapod crustaceans, such as oxidative stress in *Callinectes sapidus* (Schock et al., 2010), *Procambarus clarkii* (Izral et al., 2018); oxidative and thermal stress in *Crassula aequilatera* (Alfaro et al., 2019); nutritional stress in *P. clarkii* (Izral et al., 2018), *Astacus leptodactylus* (Costantini et al., 2018); anthropogenic stressors such as metals and contaminants in *Orconectes virilis* (Izral, 2016); and overall health in high density stocking of *Litopenaeus vannamei* (Schock et al., 2013). Metabolomic profiles for unpaired *C. cainii* and *C. tenuimanus* have been created using untargeted liquid chromatography-mass spectrometry (LC-MS) of haemolymph (Lette et al., 2020). The two species were in different reproductive and growth stages at the time of the study; *C. cainii* had completed breeding and moulting was about to commence, whereas *C. tenuimanus* were still mating. Metabolomic profiles clearly distinguished the two species, as well as males from females and variations in metabolites were indicative of high-energy processes such as reproduction and moulting.

The current study sought to determine whether the metabolome of *C. tenuimanus* differed between the sexes, and over time, and whether variation could be attributed to pairing of the sexes during the breeding season. We identified 54 annotated metabolites and metabolomic profiles differentiated females from males. Trends over time in profile changes after pairing indicate differences in reproductive physiological processes between *C. tenuimanus* sexes and between the two marron species.

## Materials and Methods

### Experimental Design

Sexually mature Margaret River hairy marron, *C. tenuimanus* (i.e., 2+ years of age) were randomly selected from captive bred stock at the Department of Primary Industries and Regional Development (WA; DPIRD) Freshwater Research Centre in Pemberton, Western Australia (PFRC) [335 km south of Perth (34.443^*o*^S 116.034^*o*^E)] and transferred to Edith Cowan University (ECU) Joondalup Campus in Perth, Western Australia (31.751^*o*^S 115.772^*o*^E) on 5 September 2017. The animals were originally from wild populations two generations prior (DPIRD, unpublished).

Females had an average ocular carapace length of 56.64 ± 3.67 mm and males 67.94 ± 7.09 mm. Average body weights were 116.13 ± 20.43 g for females and 197.94 ± 48.05 g for males (values are means ± standard deviation of five individuals). On arrival *C. tenuimanus* were bathed in salt water (30 g/L NaCl) for 2 min (as per Langdon, 1991) to ensure the animals were healthy by removing external parasites (Jones, 1998); weighed (Mettler Toledo PB3002-S); occipital carapace length (OCL) measured using Vernier calipers to the nearest 0.01 mm; and moult staged (Burton and Mitchell, 1987). Crayfish were housed individually in aquaria (approx. 50 L) and acclimated for 8 weeks, in a climate-controlled aquarium room with a photoperiod of 10 light: 14 h dark, simulating ambient conditions, using cool daylight 36W triphosphor tubes.

Aquaria were placed randomly throughout the room, ensuring equal light exposure with visual barriers in place so animals in different tanks were not visible each other. Water was from the Perth Integrated Water Supply Scheme (conductivity 108–590 uS/cm) de-chlorinated with API^©^ Tap Water Conditioner (1 mg/20 L) aerated for at least 24 h before use. Each aquarium was aerated and filtered using airlift biofilters enclosed in hard mesh to block marron from accessing the filter medium. Before adding marron to the tanks, water was conditioned with Seachem Stability^®^ at start up and weekly for 2 weeks and then left for another 4 weeks. Each tank contained two hides made of 200 mm sections of 90 mm Australian standard stormwater grade polyvinyl chloride.

Water temperature was set to 19–20^*o*^C and controlled using a closed circulating system (max. rate 550 L/h) for each aquarium into a TECO2000 chilling unit. The substrate consisted of small gravel and shell grit to an average depth of 20 mm (Viau and Rodríguez, 2010). Water quality (temperature, pH, NO_2_, NO_3_, NH_3_/NH_4_) was maintained within recommended limits (Johnston and Jungalwalla, 2005) and 10–25% (more if water quality was low) of the water was changed weekly, including siphoning of gravel. Marron were fed three times a week, a couple of hours before darkness, to a total of 3% of their body weight weekly (Lawrence, 2007)with a mixture of Skrettings Nova ME 3 mm Marine Fish Pelletts, New Life Optimum Freshwater Flakes + Garlic and Tropical^®^ Spirulina Super Forte Granulat. Unconsumed food was removed.

The time component of the design involved pre- and post-pairing of the sexes. Day 0 marked the first date haemolymph was collected when marron were held as individuals in separate aquaria. Males and females were progressively paired over four days with a similar sized individual of the opposite sex so that each aquarium contained a mixed-sex pair. Post-pairing samples of haemolymph were collected on days 12, 18, and 34. Overall the marron had been housed as pairs for an average of 30 days.

### Haemolymph Collection and Processing

Haemolymph was collected from the ventral sinus at the base of the 5th pereiopod using a 21G needle (Leland and Furse, 2012) and processed for LC-MS as described in Lette et al. (2020). At the start of the run, a solvent blank, matrix blank and ten condition quality control samples consisting of a pooled quality control were analysed, with additional quality control samples injected after every fifth experimental sample following standard metabolomics protocols (Broadhurst et al., 2018).

Samples were analysed on a Ultra High-Pressure Liquid Chromatography pump (Dionex UltiMate 3000 RS) coupled to an Orbitrap Q-Exactive mass spectrometer (Thermo Fisher Scientific) fitted with a heated electrospray ionization probe (HESI) using a HPLC Hypersil, GOLD C18 (100 × 2.1 mm, 1.9 μM particle size column, Thermo Fisher Scientific) with an in-line filter. Details can be found in Lette et al. (2020).

### Data Processing and Statistical Analyses

Metabolites were annotated according to Lette et al. (2020) using an in-house MS/MS spectral database and the mzCloud online spectral library^1^ and scored according to the Metabolomics Standards Initiative protocol (Sumner et al., 2007). Pathway enrichment followed the reference metabolome from Metaboanalyst 4.0 (Chong et al., 2019). Principal Components Analysis (PCA) including the quality control samples, indicated very high-quality reproducibility data.

Oxidative stress was calculated as the ratio of reduced to oxidised glutathione peak areas from the C18 positive column (Schafer and Buettner, 2001).

For each identified metabolite a two-way repeated measures ANOVA was conducted to examine the effects of sex and time on metabolite concentration (*p*-values, Table 1). Significance level was accepted at <0.05. If there was a significant interaction between sex and time, then an analysis of simple main effects was performed. Correction for multiple comparisons was performed using the method described by Benjamini and Hochberg (1995) and corrected *p*-values (*q*-values) are also reported (Table 1). All identified metabolites were combined into a single data matrix and the multivariate covariance analysed using PCA (Jolliffe, 2002). Hierarchical cluster analysis (HCA) (Kaufman and Rousseeuw, 1990; Hastie et al., 2009) was then performed to assess the similarities between individual metabolite concentrations, both PCA and HCA as described in Lette et al. (2020). For this study, a Principal Component-Canonical Variate Analysis (PC-CVA) was conducted using a five principal components projection to illustrate multivariate discrimination between the clusters. Data were log-transformed prior to univariate and multivariate analyses. All statistical analyses were performed using IBM SPSS^®^ Statistics 25 software package and Matlab^®^ scripting language version R2018a (Mathworks^®^, Natick, MA, United States).

**TABLE 1:**
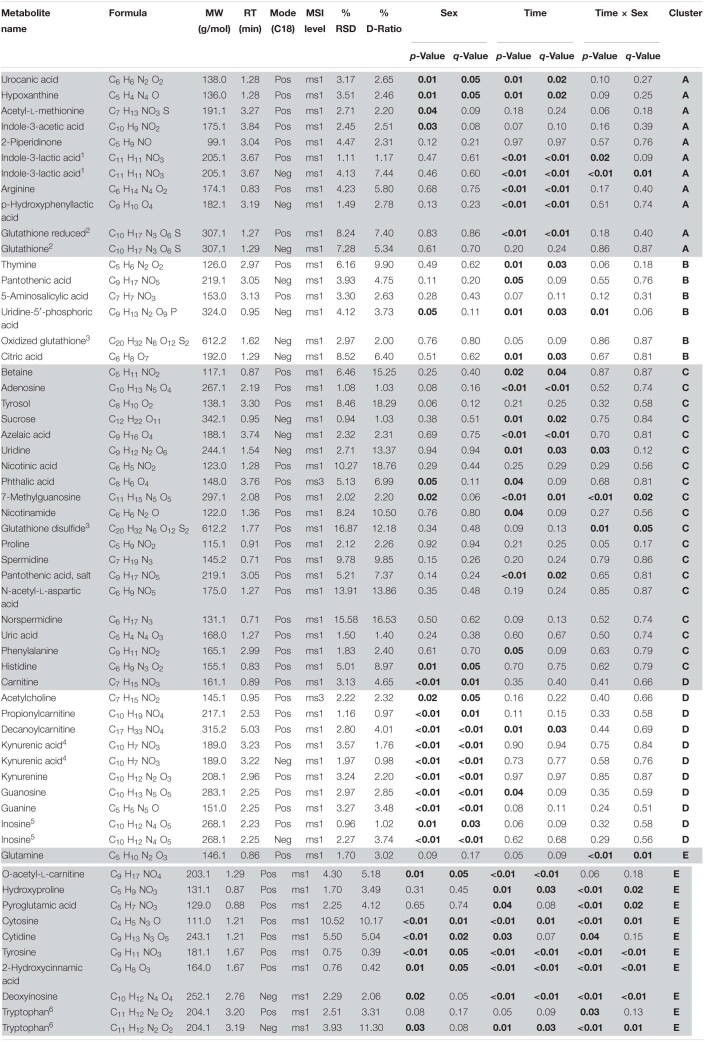
Metabolites identified in *Cherax tenuimanus* haemolymph

*Statistically significant differences between factors, based on p-value < 0.05 and q-value < 0.05 (FDR-adjusted p-values) from a two-way repeated measures ANOVA examining the effects of sex, time, and time × sex interactions on metabolite concentration, highlighted in bold. Metabolites that are listed twice (indole-3-lactic-acid, kynurenic acid, inosine, glutathione, oxidised glutathione, and tryptophan are marked with a superscript number) were identified using both the positive and negative C18 columns. MW, Molecular weight; Rt_min, Retention time in minutes; Mode, C18 column either negative or positive; MSI Level, metabolomics standards initiative level; %RSD, relative standard deviations calculated for the pooled quality control injections; %D-Ratio, false discovery rate. Metabolites in the same cluster are listed together and shaded based on hierarchical cluster analysis (Figure 2).*

## Results

**FIGURE 1:**
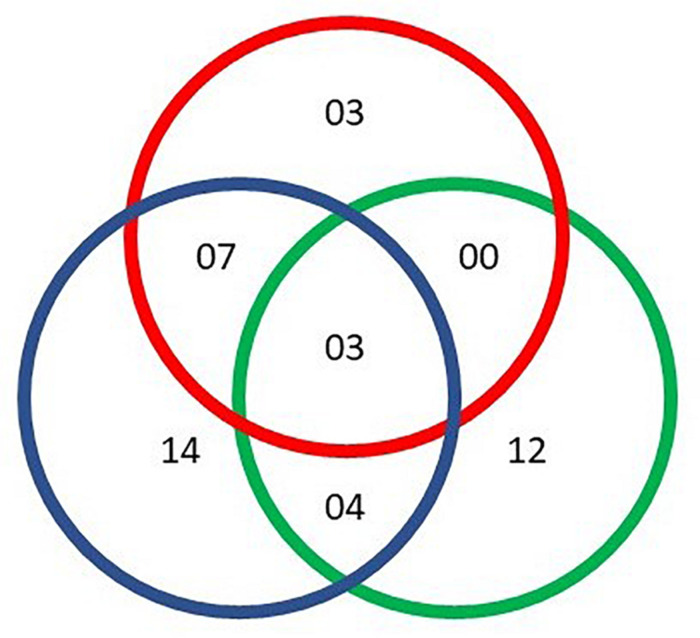
Number of metabolites identified based on sex (green), time (blue), and time × sex (red). Each metabolite showed significant changes (*q*-value < 0.05) from a two-way repeated measures ANOVA.

We identified 54 reproducible annotated metabolites including amino acids, fatty acids, biogenic amines, purine and pyrimidine metabolites and excretion metabolites (Table 1). Two-way repeated measures ANOVA corrected for multiple comparisons revealed significant differences between sexes for 19 metabolites, differences over time for 25 metabolites, and 11 metabolites had a significant interaction between sex and time (*q*-values < 0.05, FDR-adjusted *p*-values). Some metabolites were significant for more than one factor, with 14 metabolites unique to time and 12 unique to sex (Figure 1).

**FIGURE 2:**
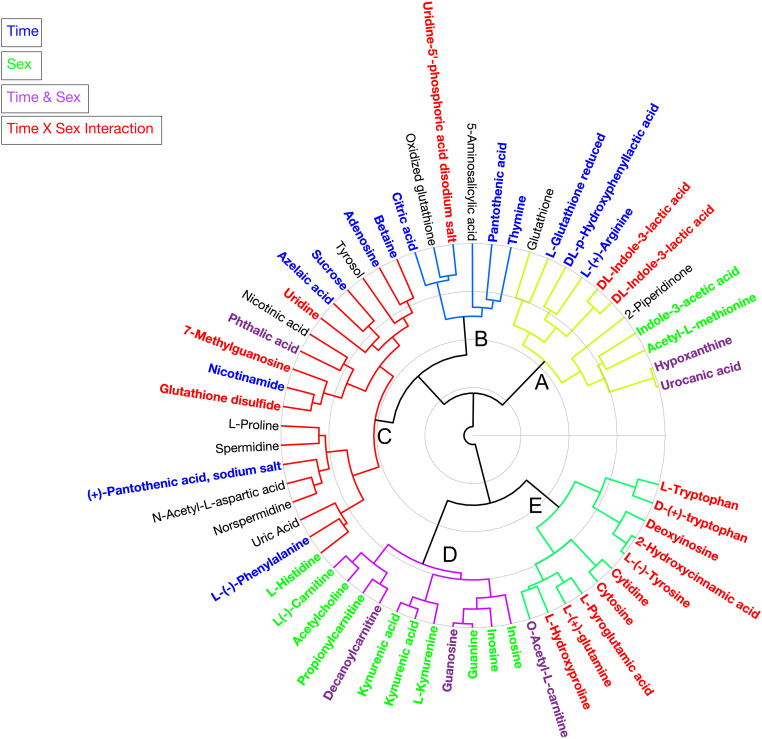
Circular hierarchical cluster analysis (HCA) dendrogram grouping individual metabolites identified in the haemolymph of *Cherax tenuimanus* into five clusters **(A–E)** based on phenotypic similarity. Colours of the metabolite name are based on the univariate analysis (*q*-value < 0.05), metabolites that are significant over time (blue), between sexes (green), time and sex (purple), time × sex interaction (red), and not significant for any factor (black). Duplicate metabolites identified in positive and negative modes including L- and D-Trp.

Hierarchical clustering analysis of the metabolites produced five clusters (Figure 2). Each cluster contained metabolites that discriminated against several factors. In cluster A metabolites increased over the duration of the experiment and are associated predominantly with energy and tryptophan metabolism but also other amino acids including glutathione. The concentrations of metabolites in cluster B tended to be higher in females but dropped for both sexes once animals were paired and then returned to original unpaired concentrations. The metabolites in cluster B were mostly pyrimidine compounds involved in metabolism, beta-alanine metabolism, pantothenate, and CoA biosynthesis. Cluster C had the most metabolites which decreased in concentration over the duration of the experiment. These metabolites represented nicotinate and nicotinamide metabolism, glutamate metabolism, glutathione metabolism, arginine and proline metabolism, methionine metabolism, aspartate metabolism, phenylalanine metabolism, histidine metabolism, and tyrosine and derivatives. Cluster D metabolites were present in higher concentrations in females and this persisted throughout the experiment. These metabolites included several carnitines (fatty acids), a neurotransmitter (acetylcholine), amino acid derivatives, kynurenic acid and kynurenine (both play a role in tryptophan and purine metabolism), guanine, guanosine, and inosine. The metabolites in cluster E showed the most striking pattern: the concentrations were higher in females throughout the experiment and remained steady, whilst unpaired males had much lower levels. However, within 12 days of pairing, concentrations in males rapidly increased to levels seen in females. These metabolites represented purine and pyrimidine, glutathione and tryptophan metabolism.

Overall, three main patterns of metabolite concentrations were identified. Differences between sexes – metabolites that were higher in females over time and this pattern was not affected by pairing (cluster D). Reactive response to the disturbance of pairing – where both sexes showed changes, with one set of metabolites returning to initial levels (transient, cluster B) and others with sustained change (non-transient, clusters A and C). Convergent response to the disturbance of pairing for males where metabolite concentrations converged with those of females (cluster E) (Table 2).

**TABLE 2:** Significant metabolite markers and their putative functions.

Metabolite	Biochemistry	Metabolic process	Details	Response
Acetyl-L-methionine	Carnitine biosynthesis	Biosynthesis	Lipid metabolism	Differences between sexes
Adenosine	Acute oxidative stress	Oxidative stress	Increased ventilation rate, heart rate and haemolymph velocity	Non-transient response to pairing
Arginine	Arginine phosphate, endogenous phosphate reserve	Stress	Escape response	Non-transient response to pairing
Betaine	Methionine and carnitine biosynthesis	Biosynthesis	Protein synthesis and lipid metabolism	Non-transient response to pairing
Carnitine		Biosynthesis	Lipid metabolism	Non-transient response to pairing
O-acetyl-carnitine	Carnitine breakdown	Mobilisation of energy stores	Deposition of yolk stress response	Convergent response to pairing
Citric acid	TCA cycle substrate	Cellular respiration	Energy metabolism	Transient response to pairing
Decanoylcarnitine propionylcarnitine	Carnitine breakdown	Mobilisation of energy stores	Deposition of yolk stress response	Differences between sexes
Glutamine	Glutamate biosynthesis	Biosynthesis	Excitatory neurotransmitter	Convergent response to pairing
Glutamine	g-Aminobutyric acid biosynthesis		Inhibitory neurotransmitter	Convergent response to pairing
Glutathione	Reduced form	Oxidative stress	Antioxidant	Non-transient response to pairing
Glutathione	Oxidised form	Oxidative stress	Antioxidant	Transient response to pairing
Indole-3-acetic acid	Indole breakdown (derived from tryptophan by gut bacteria)	Immune response	Immune response	Non-transient response to pairing
Indole-3-lactic acid	Indole breakdown (derived from tryptophan by gut bacteria)	Immune response	Immune response	Non-transient response to pairing
Inosine	Adenosine breakdown metabolomic marker	Acute stress	Fight/flight response	Differences between sexes
Kynurenic acid	Tryptophan breakdown	Metabolic rate	Decreases metabolic rate	Differences between sexes
p-Hydroxyphenyllactic acid	Tyrosine breakdown	Stress	Antioxidant produced by gut bacteria	Non-transient response to pairing
Pantothenic acid	Coenzyme A biosynthesis	Biosynthesis	Energy metabolism	Transient response to pairing
Tryptophan	Melatonin	Reproduction	Seasonal responses	Convergent response to pairing
Tryptophan	Serotonin	Reproduction	Neurotransmitter mating behaviour GSH release	Convergent response to pairing
Tryptophan	Breaks down to kynurenic acid	Metabolic rate	Antagonist to glutamate, neuroprotective reduces metabolic rate	Differences between sexes
Tryptophan	Breaks down to kynurenine which produces nicotinic acid nicotinamide	Energy metabolism	Energy metabolism	Differences between sexes
Tryptophan	NAD^+^ biosynthesis	Energy metabolism	Energy metabolism	Non-transient response to pairing
Tryptophan	Melatonin	Reproduction	Seasonal cycles	Non-transient response to pairing
Tryptophan	Serotonin	Reproduction	Gonadal Stimulating Hormone (GSH)	Non-transient response to pairing
Tryptophan	Nicotinamide	Energy metabolism	Cellular respiration	Non-transient response to pairing
Tyrosine	Receptor tyrosine kinases	Endocrine system	Growth factors	Convergent response to pairing
Tyrosine	Catecholamine neurotransmitters	Nervous system		Convergent response to pairing

**FIGURE 3:**
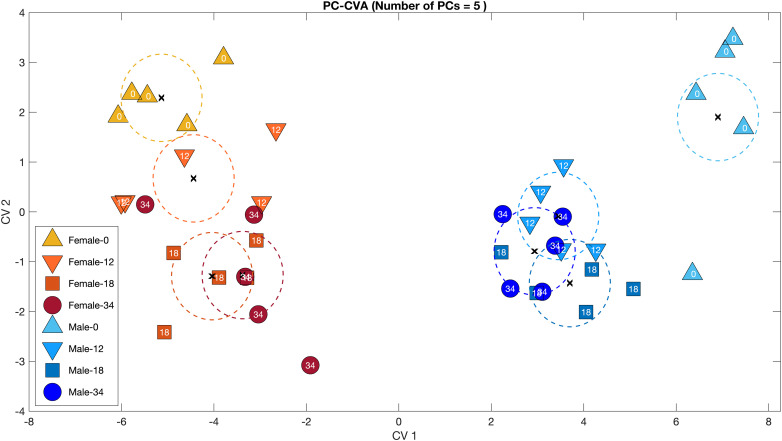
Principal component-canonical variate analysis (PC-CVA) of the relationship between metabolomic profile, sex and time from *Cherax tenuimanus* haemolymph samples. Males in cool colours (blues) and females in warm colours (reds). Numbers represent time in days and colours increase in intensity with time. At day 0, animals are unpaired and by day 12 all animals were paired and remained so for the rest of the experiment. X: mean of each group; dashed lines: 95% confidence intervals of the mean of each group. The PC-CVA model was constructed using five principal components.

Principal component-canonical variate analysis clearly shows the consistent difference between males and females, whether unpaired or paired (CV1); similar trends in both sexes over time after pairing (CV2); and the striking shift in males upon pairing (CV1 and CV2; Figure 3).

**FIGURE 4:**
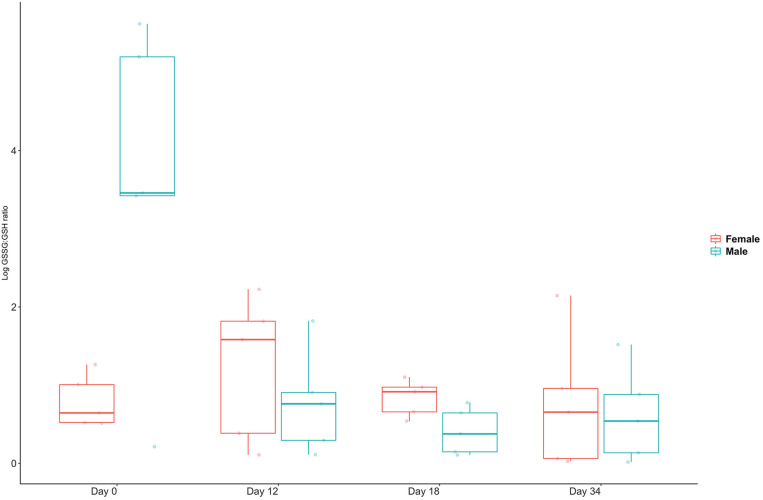
Glutathione ratios for five female and five male *C. tenuimanus* held unpaired (day 0) and paired (from between day 1–12). Box-whisker plot showing median and range of individual responses.

Oxidative stress indicated by GSSG/GSH ratios showed wide variation in unpaired males, whereas all females and paired males had low ratios (Figure 4). The ratios rose slightly in females in the immediate period after pairing and then returned to unpaired values.

## Discussion

We have previously shown that metabolomic profiles of haemolymph could discriminate between *C. tenuimanus* and its closely related congeneric *C. cainii* and between the sexes of each species (Lette et al., 2020). The current study reaffirms the second finding of distinct metabolite profiles in the sexes. The metabolic profile of *C. tenuimanus* was representative of pathways with various functions identified in other aquatic animals such as reproduction in mussel, *Perna canaliculus*, (Alfaro and Young, 2016) and in processes related to energy metabolism, osmoregulation (Rahi et al., 2018) and signalling (Hay, 2009) in decapod crustaceans.

A high proportion of the compounds identified in this study have previously been recognised in studies of other decapod crustaceans, many of which used nuclear magnetic resonance (NMR), as well as biological tissues other than haemolymph (*C. sapidus*, NMR, haemolymph (Schock et al., 2010); *L. vannamei*, NMR, whole animal, hepatopancreas, muscle, intestines (Schock et al., 2013); *A. leptodactylus*, NMR, haemolymph, muscle, hepatopancreas (Costantini et al., 2018); *P. clarkii*, NMR, hepatopancreas, gill, muscle (Izral et al., 2018); *Paralithodes camtschaticus*, LC-MS, haemolymph, muscle, hepatopancreas, and *Lithodes aequispinus*, LC-MS, haemolymph (Zacher et al., 2018). However, the ability to identify all detected metabolites is hampered by the lack of a single global database for crustaceans, which necessitates comparisons to databases created for non-crustaceans, and a lack of comprehension of the way metabolites vary between individuals of the same species, across animal groups like freshwater crayfish in particular or crustaceans in general, or even across the animal kingdom (Kuhlisch and Pohnert, 2015).

### Differences Between Sexes

The metabolomic profiles of males and females were starkly different, whether unpaired or paired (Figure 2). Individual metabolites contributing to the differences between sexes were related generally to metabolic rate and mobilization of energy stores, as well as stress and were consistently higher in females. Tryptophan here appeared to play an important role also, with breakdown products such as kynurenic acid decreasing metabolic rate by antagonistic competition with glutamate in the central nervous system, and kynurenine enhancing energy metabolism primarily in females (Janecki and Rakusa-Suszczewski, 2004). In mammals, the kynurenine pathway is the main route for tryptophan degradation (Wishart et al., 2018) and it is a metabolic pathway leading to the production of nicotinamide adenine dinucleotide (NAD^+^) from tryptophan (Wishart et al., 2018). Kynurenine is a neuroprotectant producing vitamins or cofactors such as niacin (as nicotinic acid), nicotinamide and derivatives nicotinamide adenine dinucleotide (NAD^+^ detected previously in Lette et al., 2020), and nicotinamide adenine dinucleotide phosphate (NADP), all of which participate in several energy metabolism pathways (Parthasarathy et al., 2018). Kynurenic acid is formed enzymatically from kynurenine and can be found in the intestine of mammals (Turski et al., 2013); it is an endogenous antagonist for the receptors of some stimulating amino acids such as glutamate in the central nervous system of vertebrates and invertebrates (Janecki and Rakusa-Suszczewski, 2004). The overall effect is a reduction in metabolism (Table 2).

### Reactive Responses to the Disturbance of Pairing

Metabolic responses to pairing were linked mostly to biosynthesis and stress. Energy metabolism, expressed as components of cellular respiration (Provasoli et al., 1970; Da Poian et al., 2010) was observable in transient responses. In addition, glutathione was significantly present in oxidised form, indicating oxidative stress. Glutathione occurs in reduced (GSSG) and oxidised (GSH) form and is a potent antioxidant (Halprin and Ohkawara, 1967; Lu, 2013). GSH is a redox buffer occurring in low concentrations in extracellular fluid such as haemolymph and has a role in detoxification (≤100× to −1,000× than inside the cell) (Schafer and Buettner, 2001) and protection against oxidative stress (Lu, 2013; Lavradas et al., 2014). The ratio of GSH to GSSG can be used as an indicator or biomarker for cellular oxidative stress (Alfaro et al., 2019) and can be measured in the haemolymph (Bone et al., 2015) where oxidative stress is indicated if GSSG is present at higher levels than GSH. The GSSG/GSH ratios clearly show that males experienced high and variable oxidative stress when unpaired (Figure 3). This profile was transient, as the GSSG/GSH ratios converged with those of females shortly after the disturbance of pairing. In addition, the reduced form was more prevalent in the non-transient response to pairing (Table 2). The females’ response, however, was to exhibit slightly elevated stress immediately after pairing, and then revert to lower pre-pairing ratios. This could indicate that females found pairing with a male stressful, whereas males, who may have been preparing to mate, experienced reduced stress levels once paired with a potential mate.

Reactive and convergent responses to the disturbance of pairing can also be related to biosynthetic and stress responses. The longer-term biosynthetic processes included protein synthesis and lipid metabolism and mobilisation, such as changes in tryptophan, an aromatic amino acid involved in many metabolic processes such as serotonin synthesis which is linked to the production of gonadal stimulating hormone (Alsén, 2005; Fieber, 2017; Roager and Licht, 2018). These are clear signals that the animals are preparing for mating and reproduction. At the same time, animals experienced some on-going stress from being paired as evidenced by high betaine and adenosine concentrations, seen also for *Homarus americanus* (Stegen and Grieshaber, 2001), although glutathione ratios were low in both sexes. Several bioindicators of immune responses derived from indole produced from tryptophan by gut bacteria increased in the marron, as they also do in human gut bacteria (Roager and Licht, 2018). A similar response over time has been reported in mice (*Mus musculus* C3H/He strain) where indoleacetic acid decreased 15–25 days after exposure to a stressor but then increased again after 30 days (Sun et al., 2012; Lankadurai et al., 2013). Several antioxidants were prevalent in marron, including p-hydroxyphenyllactic acid produced by gut bacteria from tyrosine and adenosine (Wishart et al., 2018).

Arginine levels increased and remained elevated when males and females were paired. Arginine is likely to increase when there is a perceived threat and potential need for an escape response by tail flipping and using arginine phosphate for energy bursts (England and Baldwin, 1983; Ellington, 2001; Morris and Adamczewska, 2002). An alternative explanation for increased and sustained arginine levels could be in response to repeated haemolymph extraction where more handling of the animal was required. Additionally, changing levels of arginine and arginine phosphate could also be indicators of preparation for moulting. Crustacean growth is incremental and moult cycles require large swings in energy metabolism and fuel storage and mobilisation (Jimenez and Kinsey, 2015). We have previously detected arginine in significantly higher levels in *C. cainii* and low levels in *C. tenuimanus* which concurs with the life stage of *C. cainii* at the time, where several males were moulting or preparing to moult (Lette et al., 2020).

Purines such as adenosine can increase ventilation rate, heart rate and haemolymph velocity in crustaceans such as *H. americanus* where it accumulates in haemolymph during hypoxia and ischaemia (Stegen and Grieshaber, 2001). Adenosine is likely to facilitate fast systemic responses to environmental stress (Stegen and Grieshaber, 2001) and in crustaceans, adenosine from muscle tissue (Stegen and Grieshaber, 2001) can work with the hormone serotonin which is released from the X-organ sinus gland complex (a neurohaemal organ) (Fieber, 2017; Reddy, 2019) in times of stress. However, changes in adenosine are difficult to assess as it has a short duration in the haemolymph; for example, Stegen and Grieshaber (2001) found it barely detectable after 2 min of infusion. Detection of adenosine in this study therefore could be an indication of extremely high levels in the animals. The breakdown product inosine is used as a bioindicator in haemolymph for the fight or flight response (Stegen and Grieshaber, 2001; Schock et al., 2013). Previously we have shown that *C. tenuimanus* females had the highest levels of adenosine and inosine of the two species, and higher than the males of both species (Lette et al., 2020).

Various breakdown products of carnitine, including decanoylcarnitine and propionylcarnitine contribute to yolk deposition and stress responses, respectively, and are used for high energy processes such as reproduction and moulting (Jimenez and Kinsey, 2015). Lipids are stored in the hepatopancreas in both sexes and are at their lowest levels during moulting (Hasek and Felder, 2006). Not only are lipids important as an energy source but they are also stored by females as lipid droplets in ovaries and other tissues in preparation for reproduction (Jimenez and Kinsey, 2015) necessary for reproductive success (Li et al., 2010). Energy reserves in the hepatopancreas are continually shifted to the ovaries via haemolymph in order to stimulate maturation of the ovaries in *Cherax quadricarinatus* but lipids from the diet are also required to maintain the needs of the ovaries (Hasek and Felder, 2006; Li et al., 2010). As well as indicating greater energy requirements in preparation for reproduction, increasing levels of carnitines can also be a response to manage stress in crustaceans (Schock et al., 2013).

Overall, females tended to have higher levels of stress bioindicators than males, and were mobilising lipid stores to deposit yolk in preparation for mating. High stress levels could have been as a result of the presence of another animal in the environment (males) as well as the energy demands of reproduction. Additionally, mating can also be dangerous for females if males are overly aggressive (Huxley, 1906). All of these responses (reduced metabolism, use of lipid stores, elevated stress) could indicate females were (a) spending less time foraging due to the presence of the male, (b) developing eggs, (c) preparing to hide while they care for the eggs after mating.

### Convergent Response to the Disturbance of Pairing for Males

The metabolites that underwent statistically significant changes in males were all lower in four out of the five unpaired males, and then converged after pairing with the concentrations found in both unpaired and paired females (Figure 3 and Table 2). These metabolites form a pattern of preparation for mating. Carnitines indicated mobilisation of energy stores, and/or stress. Several significant metabolites are precursors for neurotransmitters, glutamine, tyrosine and tryptophan, and/or growth factors, tyrosine, reproductive hormones, tryptophan.

Tryptophan showed a dramatic increase in males after pairing. As an essential amino acid, it must be obtained through diet and is one of the least abundant amino acids (Roager and Licht, 2018). Tryptophan is a precursor to the hormone melatonin (detected in our previous study, Lette et al., 2020, but not this study) and the neurotransmitter serotonin (also known as 5-hydroxytryptamine or 5-HT, not detected, Table 2) (Parthasarathy et al., 2018). Serotonin and dopamine are biogenic amines derived from tryptophan that can act as neurotransmitters in crustaceans. Serotonin has a role in determining mating behaviour in *H. americanus* (Kulkarni and Fingerman, 1992; Nagaraju, 2011), promoting ovarian maturation in crustaceans (Fieber, 2017; Reddy, 2019), stimulating the release of gonad-stimulating hormone in females *P. clarkii* (Sarojini et al., 1995), and indirectly in males releasing gonad-stimulating-factor, which results in the initiation of testicular development (Sarojini et al., 1993; Nagaraju, 2011). Serotonin was not detected in this study but as a neurotransmitter it may not normally be present in the haemolymph in crayfish. The presence of tryptophan could therefore indicate serotonin production for the release of gonad-stimulating hormone which in males may be in response to the presence of a female, whereas females appear to produce this independently of the presence of males. Dopamine was not detected in the haemolymph in this study, but some dopamine metabolites were found in the haemolymph of both marron species previously and were significantly different between species (3-O-methyldopa and phenylalanine), had an interaction between sex and species (homovanillic acid), or were not significantly different (L-dopa) (Lette et al., 2020).

Several of the metabolites that changed in males have roles in communication (or are precursors to those that do). Glutamine is a precursor for the neurotransmitters glutamate and g-aminobutyric acid (GABA), which are excitatory and inhibitory neurotransmitters, respectively (Wishart et al., 2018). Both glutamine and GABA were present in higher levels in female *C. tenuimanus* compared to *C. cainii* and males of both species in our previous study (Lette et al., 2020). Glutamate is the primary neurotransmitter in arthropods (Smarandache-Wellmann, 2016) (detected in Lette et al., 2020), and is also known to increase the metabolic rate of some invertebrates (*Abyssorchomene plebs*, an Antarctic amphipod) where glutamic acid becomes a source of energy (Janecki and Rakusa-Suszczewski, 2004). Tyrosine is part of signal transduction processes (receptor tyrosine kinases) and some of its secondary metabolites are neurotransmitters and related compounds (L-dopa, adrenaline and noradrenaline) and animal pigments (Parthasarathy et al., 2018), all of which are important for communication and as a response to the presence of a potential mate (Table 2).

These responses by the males to pairing were striking and fast, indicating the time was right for mating. And like other crayfish, the males needed the presence of a mate to complete reproductive readiness (Morrissy, 1970; Huner, 1994). Females, in contrast, appeared to prepare for reproduction in the absence of a male.

Whilst collecting haemolymph creates a stress through handling and insertion of a syringe, and has the potential to mask other effects, the amount of haemolymph collected [1,200 μL weekly, approximately 2.5% of total estimated haemolymph (Leland and Furse, 2012)] should have been rapidly replaced or adjusted through biological processes (Greco et al., 1986; Leland and Furse, 2012). The animals in the trial appeared to remain well as they fed and maintained weight during the 5-week experimental period. The extremely low GSSG/GSH ratios also indicate that the marron in the experiment were not under any oxidative stress. As well as using glutathione to indicate oxidative stress (Bone et al., 2015), alanine, glutamate, acetoacetate, succinate and trehalose can also indicate oxygen stress in laboratory studies (Izral et al., 2018). Of the listed compounds, only glutamate was detected in our study, but the other metabolites may be detectable with other LC-MS columns or targeted assays for future studies.

Amino acids such as glutamine, isoleucine, leucine, lysine, valine and betaine in tail muscle of the North American freshwater crayfish *P. clarkii* could diagnose stress when food is limited (Izral et al., 2018). Glutamine, betaine and some forms of lysine were identified and changed over time in the current study. If this change were due to food stress it may relate to differences in feeding behaviour when males and females are together, or it may represent responses to stress more generally. Therefore, these too warrant consideration as useful biomarkers.

### Implications for *ex situ* Conservation

We have identified potential biomarkers by analysing the metabolome of paired *C. tenuimanus* over a 5-week period and detecting differences in metabolites between the sexes, over time, and due to sex and time interactions. We can now generate hypotheses about how environmental conditions may affect the metabolome and investigate this via targeted metabolites, for example, research into stocking density or temperature for marron or other *Cherax* species can target metabolites identified in this study and Lette et al. (2020).

This laboratory study indicated that the crayfish metabolome is capable of diagnosing specific environmental stressors as it is fast responding and sensitive to subtle changes in the organism and its aquatic environment. Understanding the physiology of an animal and having the ability to measure metabolic stress and changes related to reproduction that are otherwise not detectable has the potential to greatly improve *ex situ* conservation and aquaculture practices.

Only recently recognised as separate species, the highly restricted *C. tenuimanus* and its widespread sister-species *C. cainii* have experienced introgressive hybridisation, with hybrids producing fertile offspring (Austin and Ryan, 2002; Duffy et al., 2014; Guildea et al., 2015); hence, it was assumed that they require similar conditions for successful breeding. *C. cainii* has been a popular aquaculture species with a long history of captive breeding in Western Australia (Morrissy, 1970, 1979; Morrissy et al., 1990; Huner, 1994; Lawrence, 2007), and requirements for captive breeding (such as food, water quality, environmental parameters, stocking densities, etc.) are well-known. However, in captive breeding situations (i.e., in ponds) the reproductive success of *C. tenuimanus* has been much lower compared to *C. cainii* with numbers of eggs per female, and the percentage of females with eggs, larvae, or juveniles often very low (compared to *C. cainii*), and it is not known whether *C. tenuimanus* are relatively poor spawners with lower fecundity naturally or if there are issues with the captive breeding practices. It is clear from this comparison that assumptions about the transferability of methods from one species to another may not be appropriate, despite the phylogenetic and morphological similarity of the species.

Hybridisation between these congeneric marron species has been confirmed (Austin and Ryan, 2002; Bunn et al., 2008; Kennington et al., 2014; Guildea et al., 2015), but it has been suggested that there are partial reproductive barriers present as levels of introgression are lower than would be predicted under random mating (Guildea et al., 2015). The partial reproductive barriers could be explained by the difference in timing of breeding seasons: although the mating seasons of both species overlap, *C. cainii* mate and reproduce earlier in the season than *C. tenuimanus*. *C. cainii* males therefore may be larger, due to earlier emergence of juveniles and access to resources, than *C. tenuimanus*. This, with the earlier breeding season of *C. cainii* is likely to prevent *C. tenuimanus* males access to potential mates and furthering the decline of the wild *C. tenuimanus* population (Guildea et al., 2015).

The current study provides clues about the physiological processes that may underpin this displacement mechanism. Our data show that female *C. tenuimanus* maintained constant levels for many metabolites regardless of pairing whereas male *C. tenuimanus* profiles changed in response to exposure to a female, suggesting that the presence of a reproductively active female may be required for the male to become ready to mate. It is hypothesised that females are cued for final reproductive preparations by environmental conditions and once reproductively ready the females provide a chemical signal or chemical-plus-visual signals (Acquistapace et al., 2002) that trigger males to undergo their last stages in preparing to mate. This would also explain our observations that some *C. tenuimanus* did mate in aquaria but those males kept unpaired did not seem ready until placed with a female (see Lette, 2020). The more aggressive and abundant *C. cainii* males will generally outcompete *C. tenuimanus* males for access to the available *C. cainii* females; this issue would be exacerbated if the *C. tenuimanus* males had not yet completed their own intrinsic preparation when the cue came from the *C. cainii* females. For the period where the reproductive receptiveness of both species’ females overlap, *C. cainii* will continue to interfere with attempts by the male *C. tenuimanus* to mate and some hybrids would be produced. Particularly if *C. tenuimanus* densities (in the wild) are so low that finding females is problematic, this pseudo-competition may also be taking place indirectly, resulting in fewer conspecific encounters and mating between *C*. *tenuimanus* individuals (Guildea et al., 2015). By the time the *C. cainii* females have finished their reproductive cycle, and *C. tenuimanus* females have exclusive access to the males, the males may have reached the end of their reproductive cycle.

Another factor limiting the reproductive success of *C. tenuimanus* is that female crayfish in the family Parastacidae appear to mate only once per season (McLay and van den Brink, 2016) as they extrude eggs within hours of mating (see Lette, 2020). In contrast, species in other families of freshwater crayfish use a strategy referred to as multiple mating, such as Astacidae [*Austropotamobius italicus* (Galeotti et al., 2007, 2012)] and Cambaridae [*Orconectes placidus*, *P. clarkii* (McLay and van den Brink, 2016)], where females will receive spermatophores from multiple males and extrude eggs days or weeks after mating. This polyandrous strategy does pose a risk of injury to females during mating (Huxley, 1906; Galeotti et al., 2007), but also the benefits in terms of egg fertilisation success with the amount and quality of sperm released (Galeotti et al., 2007). It has been confirmed that male *Cherax* can have multiple ejaculations (potentially of varying quantity/quality) over a season with potential mates (R. Duffy, unpublished), as seen also in *A. italicus* (Rubolini et al., 2006; Galeotti et al., 2012; McLay and van den Brink, 2016) and shown in simulation experiments with *Cherax destructor* (Jerry, 2001). This highlights two problems for *C. tenuimanus;* firstly, if there is only one chance for a *C. tenuimanus* female to mate and if *C. cainii* males have an advantage and can mate repeatedly they are likely to do so before her conspecific mates. The other depends on the quantity and quality (viability) of sperm released while mating, as if a *C. tenuimanus* male has low quality or volume of sperm (which may be connected to being triggered too early by a *C. cainii* female) this could also impact the reproductive success of *C. tenuimanus*.

Improving the outcomes of captive breeding programs for highly endangered species is difficult; the limited availability of specimens means working with low sample numbers or few replicates, which affects the resolving power of data. Furthermore, *ex situ* conservation is expensive. For these reasons, any tools that can quickly and reliably contribute to the knowledge of the complex processes involved in reproduction and captive breeding could be incredibly valuable. In this study we have demonstrated that the metabolome of *C. tenuimanus* provides bioindicators related to reproduction and stress that can enhance conservation through captive breeding.

## Data Availability Statement

This data is available at the NIH Common Fund’s National Metabolomics Data Repository (NMDR) website, the Metabolomics Workbench, https://www.metabolomicsworkbench.org where it has been assigned Project ID PR000001. The data can be accessed directly via it’s Project doi: 10.21228/M8159B. This work is supported by NIH grant U2C-DK119886.

## Author Contributions

EL conceived ideas and co-designed the study, conducted experiments/data collection, data analysis and interpretation, and co-wrote manuscript. QB, PH, DB, and RD conceived ideas and co-designed the study. QB, PH, RD, and AK co-supervised. NL designed the untargeted metabolomics analysis and supervised the LC-HRMS data acquisition. MB developed the analytical chemistry sampling, preparation, and loading protocols. RD provided funding and access to animals. AK assisted with data interpretation and co-wrote the manuscript. All authors contributed to manuscript revision, read and approved the submitted version.

## Conflict of Interest

The authors declare that the research was conducted in the absence of any commercial or financial relationships that could be construed as a potential conflict of interest.

## Footnotes

1 https://www.mzcloud.org/
